# A simultaneous bilateral quadriceps and patellar tendons rupture in patients with chronic kidney disease undergoing long-term hemodialysis: a case report

**DOI:** 10.1186/s12891-020-03204-6

**Published:** 2020-03-19

**Authors:** Zhengbo Tao, Wenbo Liu, Weifeng Ma, Peng Luo, Shengpeng Zhi, Renyi Zhou

**Affiliations:** Department of Orthopaedics, First Hospital of China Medical University, 155 Nan Jing North Street, Shenyang, 110001 Liaoning China

**Keywords:** Quadriceps tendon, Patellar tendon, Rupture, Haemodialysis, Chronic kidney disease

## Abstract

**Background:**

The incidence of rupture of the quadriceps or patellar tendon s is low, especially that of bilateral quadriceps tendon rupture, and it is generally considered a complication secondary to chronic systemic disorders. We report two rare cases of simultaneous bilateral tendon rupture affecting the extensor function of the knee in patients with chronic kidney disease who have been treated with long-term haemodialysis.

**Case presentation:**

Two young males with a history of chronic kidney disease who were being treated with long-term haemodialysis presented to our hospital with clinical signs of disruption of the extensor mechanism of the knee. One patient was diagnosed with bilateral quadriceps tendon rupture, and the other patient had bilateral patellar tendon rupture. They underwent surgical repair of the tendons, and their knees were actively mobilized during physiotherapy.

**Conclusion:**

Bilateral quadriceps or patellar tendons rupture is a rare occurrence in patients with chronic kidney disease who are being treated with long-term haemodialysis. Timely surgical treatment and scientific physiotherapy can lead to good recovery of knee joint function.

## Background

The disruption of the extensor mechanism of the knee is commonly caused by fractures of the patella; the incidence of rupture of the quadriceps or patellar tendons is low [1], especially that of simultaneous bilateral quadriceps tendon rupture, which only accounts for less than 5% of all quadriceps tendon ruptures. From January 1, 2018 to June 30, 2019, our group treated a total of 126 patients with quadriceps or patellar tendons rupture, and there were 7 patients with chronic kidney disease undergoing long-term haemodialysis. There were only two cases of simultaneous bilateral quadriceps or patellar tendons rupture, and both of them had undergone long-term haemodialysis. So in this case series, the incidence of simultaneous bilateral quadriceps or patellar tendons rupture was about 1.59% (2/126), but the incidence will reach 28.57% (2/7) in patients with chronic kidney disease undergoing long-term haemodialysis. In general, it is considered a complication secondary to chronic systemic disorders, such as systemic lupus erythematosus (SLE), chronic kidney disease (CKD), hyperparathyroidism and psoriasis. Recent studies indicate that bilateral quadriceps or patellar tendons rupture can occur in patients with uraemia undergoing regular haemodialysis. Their bilateral quadriceps or patellar tendonss can rupture spontaneously or due to trauma [2–5]. Although the exact mechanism is unclear, most researchers believe that secondary hyperparathyroidism caused by regular haemodialysis plays an important role in the pathogenesis of tendon rupture [3–5]. Early surgery for tendon rupture produces better results than delayed surgery [6–9]. Here, we present two rare cases of simultaneous bilateral tendon rupture in patients with CKD who were being treated with long-term haemodialysis.

## Case presentation

### Case 1

A 33-year-old male presented to the First Affiliated Hospital of China Medical University with leg pain and was unable to extend his knees. His injury occurred when he suddenly twisted his body. He had a known history of chronic kidney disease and had received regular haemodialysis in our hospital for 9 years.

The physical examination showed that both of his knees had massive suprapatellar swelling and that there was apparent depression of the soft tissue above the bilateral patella. Both knees were very soft and could touch the area over the suprapatellar region, and the patella could move freely over a larger range of motion than normal. Upon palpation, the continuity of the quadriceps tendons had defects. In addition, the patient could not complete knee extension adequately.

An X-ray of the knees showed that the superior pole of the patella moved downward, and there were calcified deposits in both quadriceps tendons. An MRI scan showed that the continuity of both quadriceps tendons was interrupted at the superior pole of the patella. The patient was diagnosed with bilateral quadriceps tendon rupture.

**Fig. 1:**
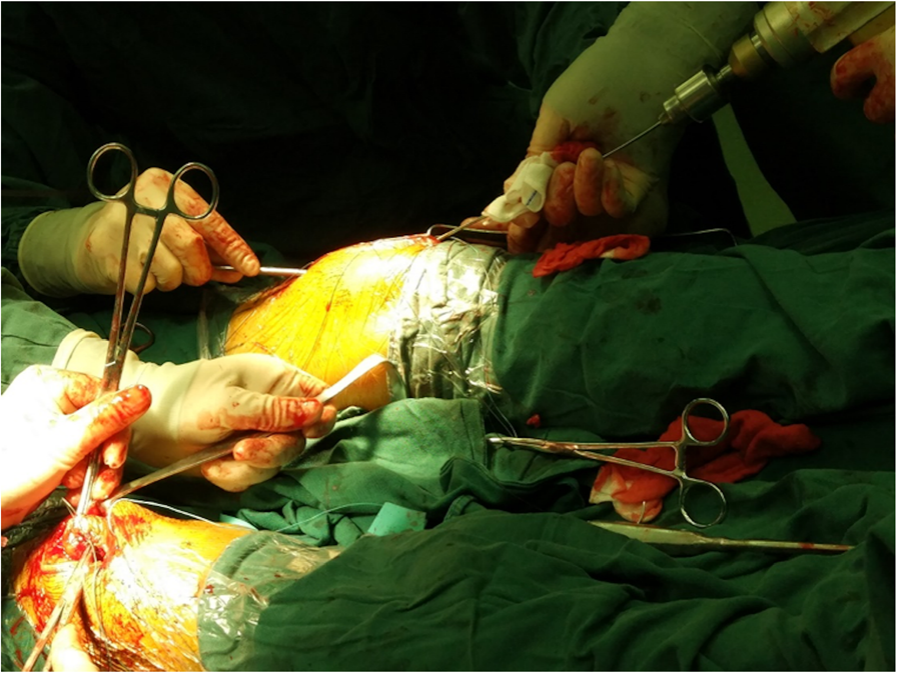
Patient undergoing bilateral quadriceps tendon reconstruction at the same time

The patient underwent bilateral quadriceps tendon suture surgery under combined spinal and epidural anaesthesia (Fig. 1). In the operating room, the patient was placed in the supine position. The surgical area was cleansed and draped, and the same operation was performed on both legs. A longitudinal incision of approximately 8 cm was made on each knee to expose the bilateral quadriceps tendon and the proximal patella. After the haemorrhage and blood clots were cleaned, complete rupture of the quadriceps tendon at the patella superior pole extending to the quadriceps muscle was observed, and the tissue stump appeared dark brown. The surface of the patella was smooth, with no residual tendon tissue attached. We used a spherical burr to roughen the upper pole of the patella after cleaning the inactivate quadriceps tendon tissue and implanted two anchors into the patella. Krackow’s suture technique was used to place two pairs of non-absorbable and heavy sutures through the quadriceps tendon. The knee could be bent to 120° during the operation without straining the sutured tendon.

**Fig. 2:**
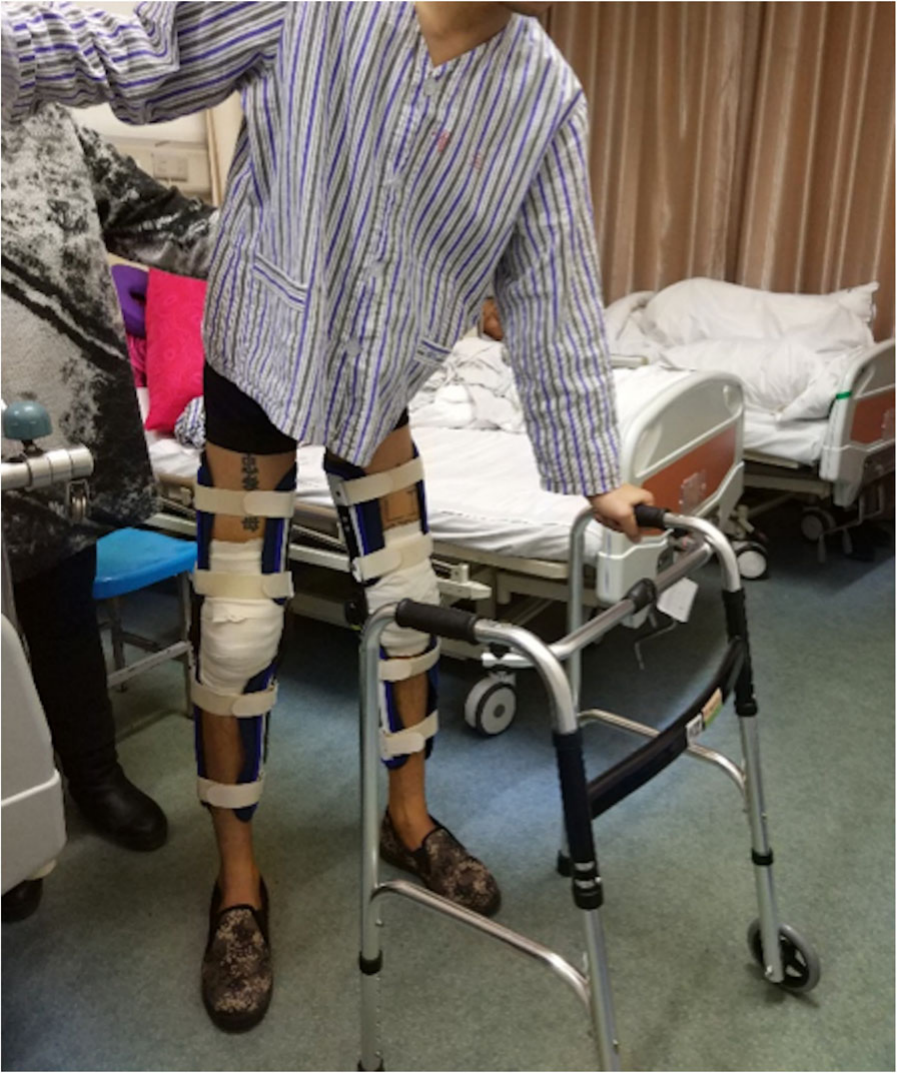
Two days after surgery, the patient wore an adjustable knee brace of 0–30 ° and stood down with the help of a walker

After an extended knee fixation period of 4 weeks, continuous passive exercise was performed, and auxiliary physical therapy was administered to exercise the extensor function (Fig. 2). We used a flexion and extension angle of < 60° to improve the range of motion. At 8 weeks after the operation, the joint mobility of the patient was 0–130°. At the 12-month follow-up visit, he had regained full mobility of the knee joint and was able to take part in various activities in daily life.

### Case 2

A 34-year-old male with a history of CKD had been undergoing long-term haemodialysis treatment for 11 years. He presented with clinical signs of ruptured patellar tendons after falling down the stairs. His physical examinations revealed that his left knee was swelling and could not perform active knee extension. In addition, a deep gap existed under his left patella.

**Fig. 3:**
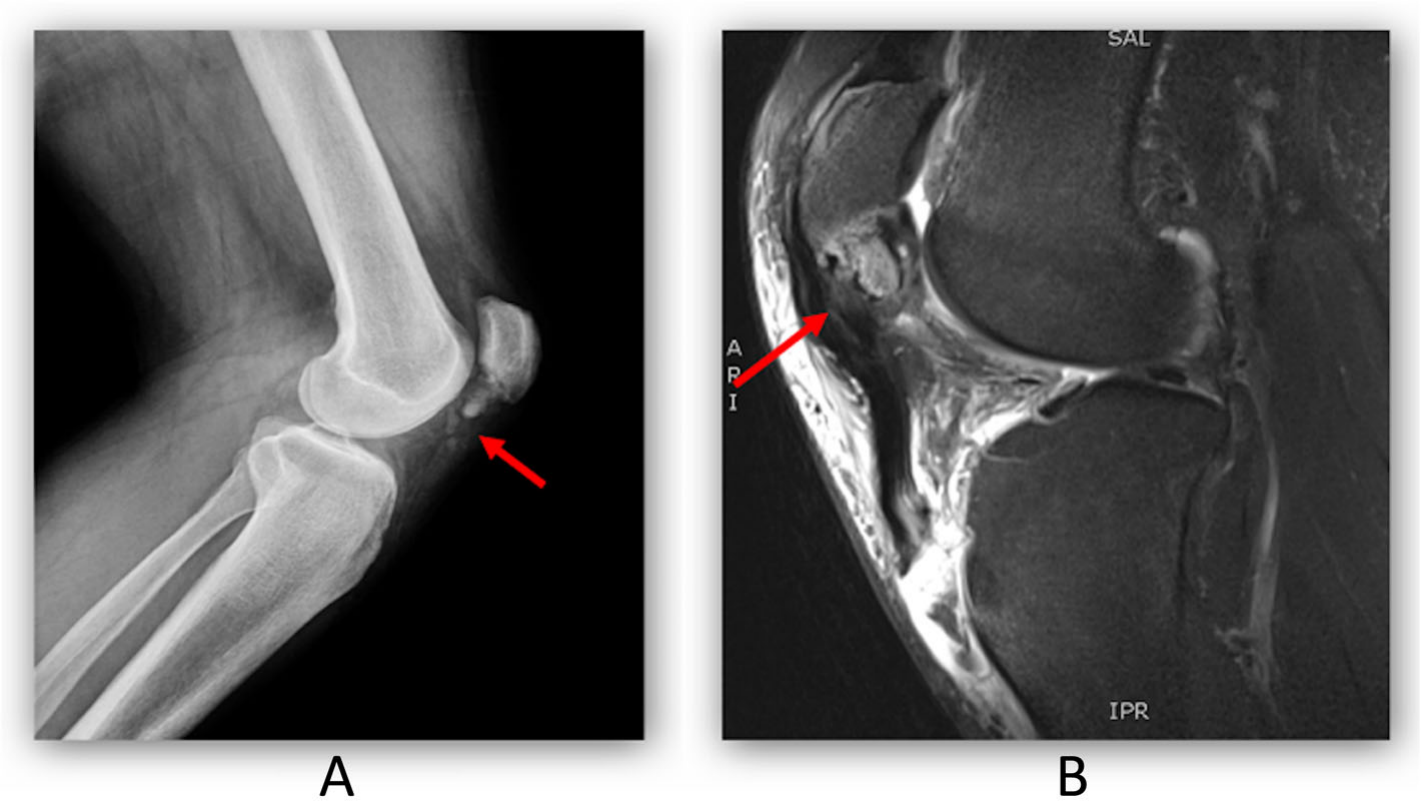
**a** Knee DR suggested avulsion fracture of the subtarsal pole and metatarsal position up; **b** Knee MR T2WI indicated rupture of patellar tendon, avulsion fracture of subtarsal pole

The diagnosis was confirmed by X-ray and MRI examinations of the knee. The patellar tendon tore at the patellar inferior pole, resulting in patellar avulsion fracture (Fig. 3).

The patient underwent surgery to repair the patellar tendon. The patient was transferred to the operating room for spinal anaesthesia. He was supine on the operating table. First, we made a 5 cm longitudinal incision overlying the patellar tendon and distal patella. After the subcutaneous tissues were removed, the patellar tendon was exposed. It was completely torn apart from the distal pole of the patella. We used a spherical burr to fresh the lower pole of the patella and Krackow’s suture technique to place two pairs of non-absorbable and heavy sutures through the quadriceps tendon. The patient started physical therapy within 1 week after surgery, and crutches and knee braces were used for walking until the patient regained sufficient strength in the patellar tendon. To maintain the stability of the repaired tendon, it should be emphasized that the flexion motion of the knee joint must gradually increase by 10–15 degrees every week. Two months after surgery, the knee’s range of motion increased to near normal, 0°-90°, and the patient could walk with full weight-bearing without axillary crutches. Finally, the patient regained full mobility of the knee joint and could take part in normal activities in daily life.

## Discussion and conclusion

Quadriceps tendons and patellar tendons are tough components of the knee that enable extension, and their structure and biomechanical characteristics make them sufficiently stable to protect the knee from forces. Therefore, ruptures of these tendons are uncommon injuries that occur mainly in middle-aged and older aged men. Simultaneous rupture of bilateral quadriceps tendons is rare, accounting for less than 5% of all cases and it’s much more difficult to repair than a unilateral tendon rupture [10]. In addition, some research indicated there was an association between patella spurs and quadriceps tendon ruptures, we should pay more attention when the knee radiographs found a patella spur [11]. In 1949, the first case of bilateral quadriceps tendon rupture with chronic renal failure was reported by Steiner and Palmer [2]. Some similar cases were subsequently reported, we collected and summarized them into a table [3, 4, 12–24] (Table 1). We searched these case reports on Pubmed and the search strategy was presented as follows: TI = (simultaneous bilateral quadriceps tendon rupture, hemodialysis) AND Language = English. Although the exact mechanism is unclear, most researchers believe that secondary hyperparathyroidism or other hormonal imbalance caused by regular haemodialysis has an important impact on the pathogenesis of tendon rupture [25]. Vitamin D deficiency and secondary hyperparathyroidism may result in subperiosteal bone resorption, which can weaken the firmness between the quadriceps tendon and patella [26, 27].

**Table 1:** Previous cases of quadriceps and patellar tendon ruptures during haemodyalisis

	Study	Year	Cases	Content
1	*Damir Matokovic* et al. [3]	2010	1	Spontaneous concurrent bilateral rupture of the quadriceps tendons in a patient with chronic renal failure
2	*Yong Hwan Kim* et al. [4]	2006	1	Spontaneous and simultaneous rupture of both quadriceps tendons in a patient with chronic renal failure
3	*Chris H. L. Lim* et al. [12]	2016	1	Simultaneous bilateral quadriceps tendon ruptures in a patient with chronic renal insufficiency
4	*Weiqian Wu* et al. [13]	2019	1	Simultaneous spontaneous bilateral quadriceps tendon rupture with secondary hyperparathyroidism in a patient receiving hemodialysis
5	*Maofeng Gao* et al. [14]	2013	1	Simultaneous bilateral quadriceps tendon rupture in a patient with hyperparathyroidism undergoing long-term haemodialysis
6	*Nisar A Wani* et al. [15]	2011	1	Bilateral spontaneous quadriceps tendon rupture in a woman who has CKD.
7	*Jin Hee Park* et al. [16]	2013	1	Spontaneous and serial rupture of both Achilles tendons associated with secondary hyperparathyroidism in a patient receiving long-term hemodialysis
8	*Chusheng Seng* et al. [17]	2015	2	Spontaneous disruption of the knee extensor: the first patient had connective tissue disease and long-term steroid use and the second patient had end-stage renal failure with tertiary hyperparathyroidism and was on haemodialysis
9	*Adnan Kara* et al. [18]	2013	2	Osteotendinous repair of bilateral spontaneous quadriceps tendon ruptures with the Krackow technique in two patients with chronic renal failure
10	*Byung Soo Kim* et al. [19]	2012	1	Simultaneous bilateral quadriceps tendon rupture in a patient with chronic renal failure
11	*Yunseok Lee* et al. [20]	2011	1	Simultaneous bilateral quadriceps tendon rupture in a patient with chronic renal failure
12	*Giuseppe Grecomoro* et al. [21]	2008	1	A 48-year-old man with chronic, spontaneous and simultaneous quadriceps, and contra-lateral patellar tendon rupture
13	*Cemil Kayali* et al. [22]	2008	1	Simultaneous bilateral quadriceps tendon rupture in a patient on chronic haemodialysis
14	*Martin Rysavy* et al. [23]	2005	1	Spontaneous and simultaneous quadriceps and patella tendon rupture in a patient on chronic hemodialysis
15	*Hasan Hilmi Muratli* et al. [24]	2005	1	Simultaneous rupture of the quadriceps tendon and contralateral patellar tendon in a patient with chronic renal failure

The kidney is an organ that plays a dominant role in bone metabolism. Patients who suffer from CKD usually have a high incidence of renal osteopathy, which is characterized by the metabolic disturbance of bone and minerals [28–30]. Uraemia patients have to rely on long-term haemodialysis to sustain life, and they usually experience calcium-phosphorus metabolism disturbance, which ultimately results in secondary hyperparathyroidism. Excessive amounts of parathormone leads to degeneration of tendon tissue, and a decline in active vitamin D receptors in the parathyroid gland cause a low level of active vitamin D, which results in ligament and tendon tissue denaturation. In addition, metabolic acidosis also causes collagen synthesis disorder [28]. Generally, these factors bring about abnormal collagen components in tendon tissues.

To prevent this kind of trauma, patients who are being treated with long-term haemodialysis should pay more attention to their daily activities. Reducing strenuous exercise and using the proper safety gear are good preventative measures. Once quadriceps or patellar tendons rupture occurs, early surgical treatment is the best choice to recover physiological function of the components involved in knee extension. Studies have suggested that early (within 2 weeks) surgery has the best clinical efficacy [6–9] because there is less fibrous cicatricial tissue and retraction during this period.

Suture anchors are a powerful and effective technique and have been used to treat quadriceps or patellar tendons rupture. In these cases, the bone and tendon are fixed with a suture anchor system (Smith & Nephew Twinfix), two anchors are implanted into the patella, and two non-absorbable sutures are placed through the tendons. Two anchors (d = 5 mm) with a depth of 2 mm below the surface of the patella are screwed in, and then, the sutures are used to fix the tendons by Krackow’s suture technique. There are some advantages of this system: (1) the suture anchor is a self-tapping screw, and it does not require a hole to be drilled during the surgery, so the risk of patellar fracture is low. (2) The suture material has good strength and histocompatibility, can support patients’ early functional training and does not easily cause rejection reactions. (3) The implanted point of the anchor is near the normal attachment of the ligaments, and the fixation of the tendons basically conforms to the physiological anatomic relationship; therefore, the natural biomechanics of the quadriceps or patellar tendons is retained. Compared with classic transosseous suture, the suture anchor system may not have a cost advantage, but this minimally invasive operation can save surgical time, recover quickly, and have better biomechanical results [31].

For the patient who suffered from bilateral quadriceps tendon rupture, the postoperative immobilization period in knee extension needed to last 4 weeks. Then, continuous passive exercise was performed, and auxiliary physical therapy was applied to exercise the extensor function. We used a flexion and extension angle of < 60° to improve the range of motion. For the patient who suffered from bilateral patellar tendon rupture, physical therapy was started within 1 week after surgery, and crutches and knee braces were used for walking until the patient regained sufficient strength in the patellar tendon. To maintain the stability of the repaired tendon, it should be emphasized that the flexion motion of the knee joint must gradually increase by 10–15 degrees every week. Two months after surgery, knee range of motion increased to near normal, 0°-90°. And if the correct exercise is performed, there is still a great chance of recovery and even participate in sports activities [32].

## Data Availability

All data generated or analyzed during this study are included in this article.

## Abbreviations

CKD: Chronic kidney disease
MRI: Magnetic resonance imaging
